# Improved time to treatment failure and survival in ibrutinib-treated malignancies with a pharmaceutical care program: an observational cohort study

**DOI:** 10.1007/s00277-020-04045-y

**Published:** 2020-06-01

**Authors:** Jeremie Zerbit, Sylvie Chevret, Sophie Bernard, Marie Kroemer, Charlotte Ablard, Stephanie Harel, Pauline Brice, Isabelle Madelaine, Catherine Thieblemont

**Affiliations:** 1 Pharmacy Department, APHP, Saint-Louis Hospital, Paris, France; 2 Department of Biostatistics and Medical Information, APHP, Saint-Louis Hospital, Paris, France; 3 Paris Diderot University, Sorbonne Paris-Cité, Paris, France; 4 Hemato-Oncology Department, APHP, Saint-Louis Hospital, 1, Avenue Claude Vellefaux, 75010 Paris, France; 5 Pharmacy Department, University Hospital Center of Besançon, Besançon, France; 6 Immuno-Hematology Department, APHP, Saint-Louis Hospital, Paris, France

**Keywords:** Ibrutinib, Leukemia, Lymphoma, Survival, Pharmacist, Education

## Abstract

Ibrutinib treatment has been shown to increase survival in patients with B cell malignancies. Real-life data suggest a large part of discontinuations are due to toxicities, impairing ibrutinib efficacy. We aimed to assess the impact of a pharmaceutical care program on the efficacy and safety of ibrutinib. This single-center, cohort, observational study enrolled patients with B cell malignancies. Patients were either assigned to the program or to receive usual care, based on physician decision. The program was conducted by clinical pharmacists specializing in oncology and included patient education for management of toxicities, adherence monitoring, interventions to reduce drug-drug interactions, and follow-up of transition from hospital to community. Between February 2014 and May 2017, we enrolled 155 patients, including 42 (27%) who were allocated to the program group and 113 (73%) to the usual care group. The effect of the program was beneficial in terms of time to treatment failure (*p* = 0.0005). The 30-month progression-free and overall survivals were significantly superior in the program group (respectively *p* = 0.002 and *p* = 0.004). Grade 3 or higher adverse events occurred more frequently for patients in the usual care group (15%) than program group (8%). A pharmaceutical care program provides a personalized environment for outpatient monitoring and control of the key risks associated with oral anticancer agents. This study shows evidence that management of ibrutinib treatment by clinical pharmacists results in significant improvement in survival and better tolerance than usual care.

**Electronic supplementary material:**

The online version of this article (10.1007/s00277-020-04045-y) contains supplementary material, which is available to authorized users.

## Introduction

In the last two decades, major advances in understanding the biology of hematological malignancies have led to significant progress in their treatment. In particular, an increasing amount of oral anticancer agents (OAA) have demonstrated remarkable outcomes in patients.

Ibrutinib is a first-in-class, once-daily, oral inhibitor of Bruton tyrosine kinase (BTK) approved for the therapy of B cell malignancies including chronic lymphocytic leukemia (CLL), Waldenström macroglobulinemia (WM), marginal zone (MZL), and mantle cell lymphoma (MCL). Ibrutinib has demonstrated marked efficacy leading to improved progression-free survival (PFS) and overall survival (OS) among patients, including those with relapsed or refractory disease, and high-risk, elderly, or comorbid patients [1–4].

Benefits of ibrutinib are impaired by its self-administration in the home setting involving challenges of any OAA with respect to monitoring of toxicity, adherence, and drug-drug interactions (DDIs).

Despite acceptable tolerance reported in clinical trials, studies evaluating the causes of ibrutinib discontinuations showed that adverse events (AEs) were responsible for 80% of discontinuations in CLL [5] and of 29% in MW [6]. Data from real-world use of ibrutinib indicated a 51% discontinuation rate due to AEs [7], the most common being atrial fibrillation, infection, pneumonitis, bleeding, and arthralgia. Reviews on OAA indicate that adherence is frequently low, below 80% of patients [8]. Management of ibrutinib dose adherence appears to be decisive. On the one hand, disadherence is associated to decreased PFS in CLL patients [9], on the other hand, dose reduction because of adverse events allows continuation without affecting PFS [10]. Ibrutinib is metabolized by CYP3A4 and co-administration with strong CYP3A4 inhibitor or inducer is not recommended, while a moderate inhibitor remains possible if absolutely necessary by reducing the dose of ibrutinib to 140 mg daily [11]. Among patients treated with ibrutinib for chronic lymphocytic leukemia, 64% have co-medications that could increase toxicity by drug interactions including 18% through CYP3A4 [12].

Managing these risks is essential to keep patients on ibrutinib long enough to achieve an overall response. Pharmaceutical care program (PCP) is a potentially attractive service to improve treatment safety and quality of oral chemotherapy [13]. This study was designed to investigate the effectiveness and safety of a PCP-based management of patients receiving ibrutinib. We hypothesized that the program could impact the PFS by both improving the adherence and reducing the occurrence of adverse events in these patients.

## Methods

### Study design and participants

We conducted a single-center prospective, cohort study to assess effects of a PCP on efficacy and safety of ibrutinib.

Eligible patients were at least 18 years old, diagnosed with chronic lymphocytic leukemia, Waldenström macroglobulinemia, mantle cell lymphoma, or other B cell malignancies with evidence of disease progression requiring ibrutinib treatment according to consensus guidelines. Exclusion criteria were the initiation of ibrutinib treatment within 6 months and the monitoring in another hospital than the study center.

Patients were enrolled at the time of ibrutinib initiation and were either assigned to start the pharmaceutical care program (PCP group) or to receive only usual care without additional monitoring (control, usual care group).

The study protocol was approved by the institutional review board (IRB-00003835) and all patients provided written informed consent for participation in the study. Data collection has been declared to the National Commission for Data Processing and Freedoms. The study was registered with the French National Agency for Medicines and Health Products Safety (number 2017-A03604-49).

### Procedures

Decision of allocation to the PCP relied on their oncologist. The PCP started in the first 10 days of ibrutinib primo-prescription. Clinical pharmacists specializing in oncology conducted pharmaceutical consultations in day hospital of hematology ward. Patients were seen in PCP every 3 months until the sixth month of treatment, then every 6 months. The PCP involved at least four pharmaceutical consultations lasting between 30 and 60 min. The pharmaceutical consultations were spaced by usual oncological consultations. Patients in the control group received usual care in routine practice including monthly oncologist consultations during 3 months then every 3 months.

The PCP was multimodal and included patient education for self-management in case of toxicities, proactive adherence monitoring, medication-related interventions to reduce drug-drug interactions, and follow-up of transition from hospital to community.

The education step started with a procedure-based evaluation of patient health literacy and fixing of goal setting. Patients were leaving the PCP when goals were met. The training for AEs self-management used take-home information and guidelines for self-management interventions in case of fever and infections, diarrhea, bleeding events, fatigue, cytopenia, and atrial fibrillations. Interventions were suggested according to the severity of toxicities, using a 4-degree scale: no change, taking medication prescribed to take as needed, consulting a general practitioner, and going to emergencies. Whatever the intervention, unless any change, the patient should call either a pharmacist or oncologist. The clinical pharmacist did a telephone follow-up for patients who required an outpatient intervention.

The education aimed to increase patients understanding of the treatment, as well as of its risks, benefits, and proper use. The program required an active role of patients to give them a greater sense of responsibility and warrant an adequate adherence. Adherence was evaluated monthly by using both patient diaries (self-evaluation) and adjudicated prescription claims from pharmacy database. All patient treated by ibrutinib were asked to rate each administration on an institutional monthly diary. The claims data method calculated the medication possession ratio (MPR) defined by the percentage of supply days received divided by the dispensing period. In case of disadherence, the cause was identified and resolved by a reinforcement of the education, a relief of the side effects, or a dose reduction, as appropriate. A familiar caregiver was identified and educated, particularly in case of physical, psychic, or socio-economical barrier.

Medication-related interventions consisted of a medication review for interaction check by clinical pharmacists. Drug interactions were identified and classified as previously described [14] and by analysis of minimum three sources for collecting drug prescriptions. In case of DDI qualified as potentially clinically relevant, interventions were proposed by the pharmacist to physicians among a drug addition, withdrawal, substitution, change of administration modality, dosage adjustment, and/or a specialized medical consultation or a biological test.

The follow-up of transition from hospital to community was performed by clinical pharmacists with a formalized transmission of information to general practitioners, community pharmacists and, when appropriate, specialist organ physicians. Community care professionals in charge of patients were informed about starting of ibrutinib and inclusion in the PCP with a standard letter describing the treatment and its monitoring in the PCP. Then, they received a report following each pharmaceutical consultation. The clinical pharmacist communicated any decision leading to change patient care, especially in case of medication-related interventions or management of an adverse effect and/or a poor adhesion.

### Outcomes

The primary efficacy outcome of the study was progression-free survival (PFS, time from treatment onset to date of progression or death from any cause, whichever occurred first). Secondary outcomes were adherence rates, DDIs, time to treatment failure (TTF), and overall survival (OS, time from treatment onset to date of death). TTF was defined as the time from treatment onset to discontinuation for any reason excluding remission, i.e., disease progression, treatment toxicity, patient preference, or death.

Safety outcomes were adverse events as measured by hematologists during usual consultations and measurement of laboratory variables. Using the NCI Common Terminology Criteria for Adverse Events version 3.0, adverse events were reported monthly and graded on a 0 to 4 scale (0, normal; 4, life-threatening).

Data were collected monthly from treatment beginning to 6 months, then every 3 months.

### Statistical analysis

A propensity score (PS) approach was used to control for observed confounding factors [15, 16]. Propensity score was defined as the patient’s probability of being allocated to the PCP group, based on the individual observed covariates. Probability was estimated using a logistic regression model with PCP exposure as the dependent variable in relation to the following baseline characteristics: disease, time to treatment, and any other factors selected as associated with the outcome in the cohort. The primary analysis was based on propensity score matching, with a 1:1 matching algorithm without replacement to match exposed and non-exposed subjects on propensity score within a caliper of 0.1 standard deviation of the logit of the propensity score. Imbalance after matching thus was carefully checked using mean standard mean differences and c-index [17]. Once all imbalances have been erased, the outcomes were compared between exposed and non-exposed patients using Cox regression fit with robust variance to account for correlations within the matched pairs [18].

Sensitivity analyses were performed to handle missing covariates. Multiple imputations of missing data was performed, using chained equations, incorporating all baseline variables of the propensity score model, as recommended [19]. Thirty independent imputed datasets were generated with propensity score estimated on each, then averaged before matching.

All tests were 2-sided, with *P* values less than 0.05 considered significant. Analyses will be performed on R 3.5.1.

## Results

### Patients

**Fig. 1:**
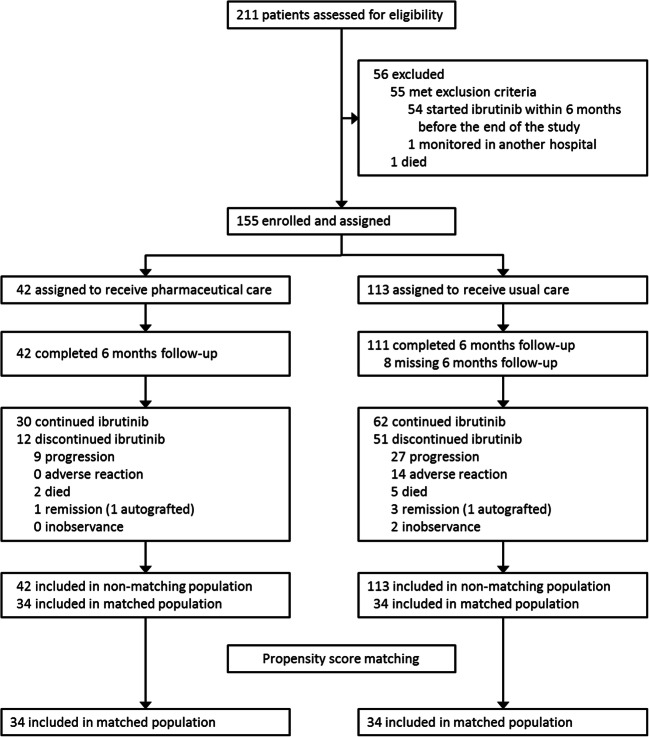
Patient flow and disposition

Between February 25, 2014, and May 9, 2017, we assessed 211 patients for eligibility, of whom 155 (73%) were subsequently enrolled. The most common reason for exclusion was initiation of ibrutinib treatment for less than 6 months. Patients were allocated to the PCP group (*n* = 42) or the usual care group (*n* = 113). Eight participants from the control group missed the 6-month follow-up (Fig. 1).

Table 1 summarizes the differences in treatment groups in the original cohort. Baseline characteristics of patients assigned either in the PCP or in the control group were not significantly different. Across the entire cohort, the median age was 70.3 years (interquartile range (IQR), 63.4–77.7 years) with 80 (51.6%) patients being 70 years of age or older (Table 1). Patients had undergone a median of two (IQR, 1–3) previous lines of therapy including 63 (40.6%) who received three or more previous lines. The median diagnostic duration before inclusion was 71.3 (IQR, 28.1–122.8) months. Main diagnoses were CLL (*n* = 80, 51.6%), mantle cell lymphoma (*n* = 32, 20.7%), and Waldenström macroglobulinemia (*n* = 23, 14.8%). Thirty-two patients had advanced-stage LLC (Rai stage III or IV), among whom 28% had the high-risk genetic features del(17p) (*n* = 29), del(11q) (*n* = 9), or *TP53* mutation (*n* = 10). Five (16%) and 18 (56%) patients had a high international prognostic scoring system (IPSS) for WM and a high MCL international prognostic index (MIPI), respectively.

**Table 1:** Characteristics of the patients at the baseline

	Original cohort		Matched cohort	
Characteristics ^a^	PCP (*n* = 42)	Usual care (*n* = 113)	PCP (*n* = 34)	Usual care (*n* = 34)
Age, years – median (IQR)	66 (60.4–73.4)	71 (64.5–78.4)	66 (59.3–73.4)	69 (62.0–77.5)
Female sex – no. (%)	16 (38)	51 (45)	11 (32)	13 (38)
Previous lines of treatment – median (IQR)	2 (1–3)	2 (1–3)	2 (1–3)	2 (1–3)
Time from last therapy, months – median (IQR)	15 (0–25)	8 (0–24)	15 (2–25)	3 (0–26)
Time from initial diagnosis, months – median (IQR)	64 (33–109)	76 (25–126)	64 (38–93)	62 (27–105)
Any comorbidities – no. (%)	29 (69)	42 (37)	N/A	N/A
Cardiovascular comorbidity – no. (%)	15 (36)	19 (17)	12 (35)	5 (15)
Other malignancies – no. (%)	9 (21)	7 (6)	3 (9)	3 (9)
Comedications – median (IQR)	4 (0–14)	5 (0–18)	N/A	N/A
Chronic lymphocytic leukemia – no. (%)	17 (40)	63 (56)	15 (44)	16 (47)
Binet stage – no. (%) ^b^				
A	0	1 (2)	0	1
B	4 (24)	4 (6)	4	1
C	13 (76)	55 (87)	11	14
Rai stage – no. (%) ^c^				
I or II	9 (53)	39 (62)	8	10
III or IV	8 (47)	24 (38)	7	6
17p deletion or TP53 mutation – no. (%)	6 (14)	29 (26)	3	6
Mantle cell lymphoma – no. (%)	16 (38)	16 (14)	11	8
MIPI – median (IQR)	6.75 (5.9–7.1)	7.6 (6.6–8.3)	6.80 (5.75–7.40)	7.30 (6.15–8.05)
High	9 (57)	9 (57)	N/A	N/A
Waldenström macroglobulinemia – no. (%)	6 (14)	17 (15)	5	3
ISSWM– no. (%)				
Low	1 (17)	3 (18)	1	1
Intermediate	1 (17)	4 (24)	1	0
High	1 (17)	4 (24)	1	1
Diffuse large B cell lymphoma – no. (%)	3 (7)	10 (9)	3	6
Hemoglobin, g/dl – median (IQR)	11.7 (10.5–13.3)	10.6 (9.4–12.1)	11.9 (10.5–13.3)	11.0 (10.0–12.2)
Platelet count, giga/L – median (IQR)	161 (102–225)	125 (61–197)	152 (101–224)	118 (58–164)
Neutrophil count, giga/L – median (IQR)	2.9 (1.9–4.5)	2.9 (1.5–5.1)	3.0 (2.0–4.6)	2.8 (2.2–4.4)
LDH > ULN – no. (%)	17 (40)	61 (55)	16 (47)	16 (47)
Initial dosage of ibrutinib – median (IQR)	420 (420–560)	420 (420–420)	420 (420–560)	420 (420–560)

*PCP* pharmaceutical care program, *IQR* interquartile range, *N/A* not applicable, *MIPI* Mantle Cell Lymphoma International Prognostic Index, *ISSWM* International Prognostic Scoring System for Waldenström macroglobulinemia, *LDH* lactate dehydrogenase, *ULN* upper limit normal

^a^No significant differences between the two groups at baseline. Percentages may not total the overall number in the category because of rounding

^b^Stage A denotes low-risk disease, stage B intermediate risk, and stage C high risk

^c^Stage I or II denotes intermediate-risk disease, and stage III or IV high risk

All the 42 patients in the PCP have benefitted of 109 pharmaceutical consultations; all participants accomplished the first step of the PCP, and 13 (31%) achieved the four consultations of the PCP. The median follow-up was 13.8 (IQR, 7.6–19.8) months and 92 (59%) patients were still receiving ibrutinib at the time of last follow-up.

**Fig. 2:**
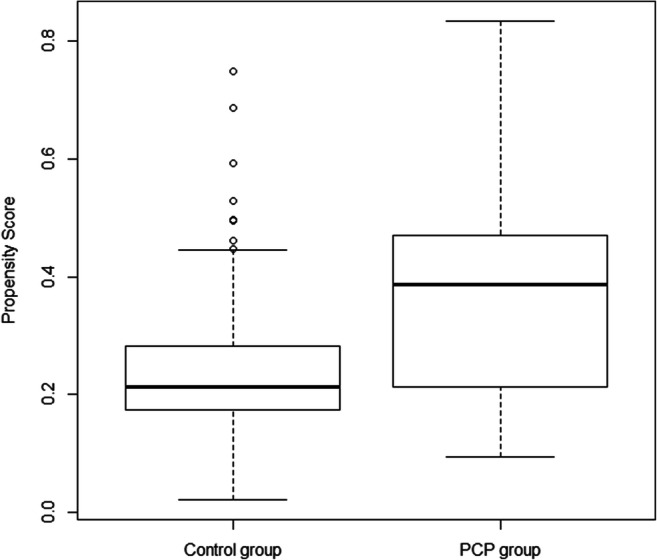
Comparison of the propensity scores on the original basis (unmatched). Difference in both treatment groups can be visualized by distribution of the propensity score, with a c-index at 0.71. PCP, pharmaceutical care program

**Fig. 3:**
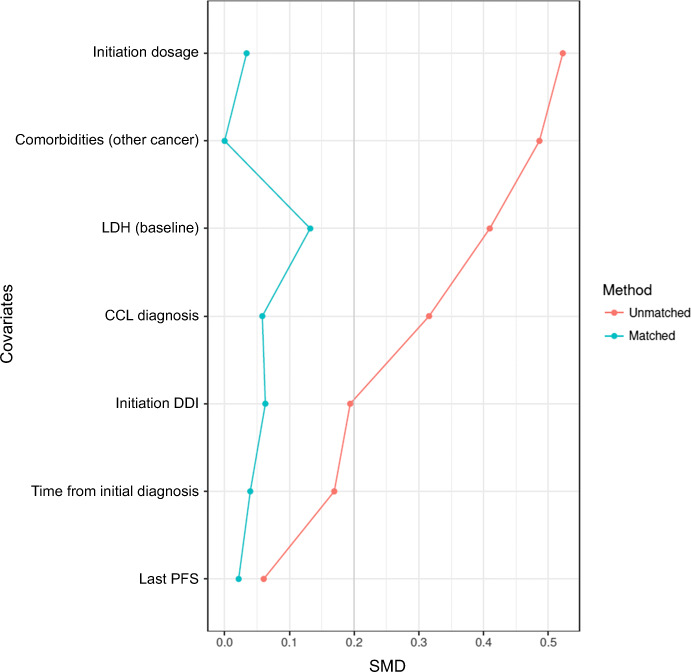
Comparison of imbalances between groups on the unmatched and matched basis. Matching without replacement on the propensity score within a caliper of 0.1 leads to holding 34 patients from the PCP group on the 42 of the original basis. After this matching, the c-index is reduced at 0.503, as well as the standardized mean differences. LDH, lactate dehydrogenase. CLL, chronic lymphocytic leukemia. DDI, drug-drug interactions. PFS, progression-free survival. SMD, standardized mean differences

The propensity score was constructed from the prognostic variables of survival, that is, CLL diagnosis, last PFS, time from initial diagnosis, initiation dosage, LDH, comorbidities (other cancer), and drug interactions measured at inclusion. At first, the model was built on complete cases, i.e., in 147 patients, including 42 patients from the PCP group. Difference in both treatment groups can be displayed by the distribution of the PS (Fig. 2), with a c-index at 0.71. Only 34 (81%) of the 42 patients from the PCP group could be matched with controls on the basis of their PS, resulting in a matched cohort with reduced imbalances in confounders (Fig. 3), as also illustrated by the c-index at 0.503.

### Efficacy endpoint

**Fig. 4:**
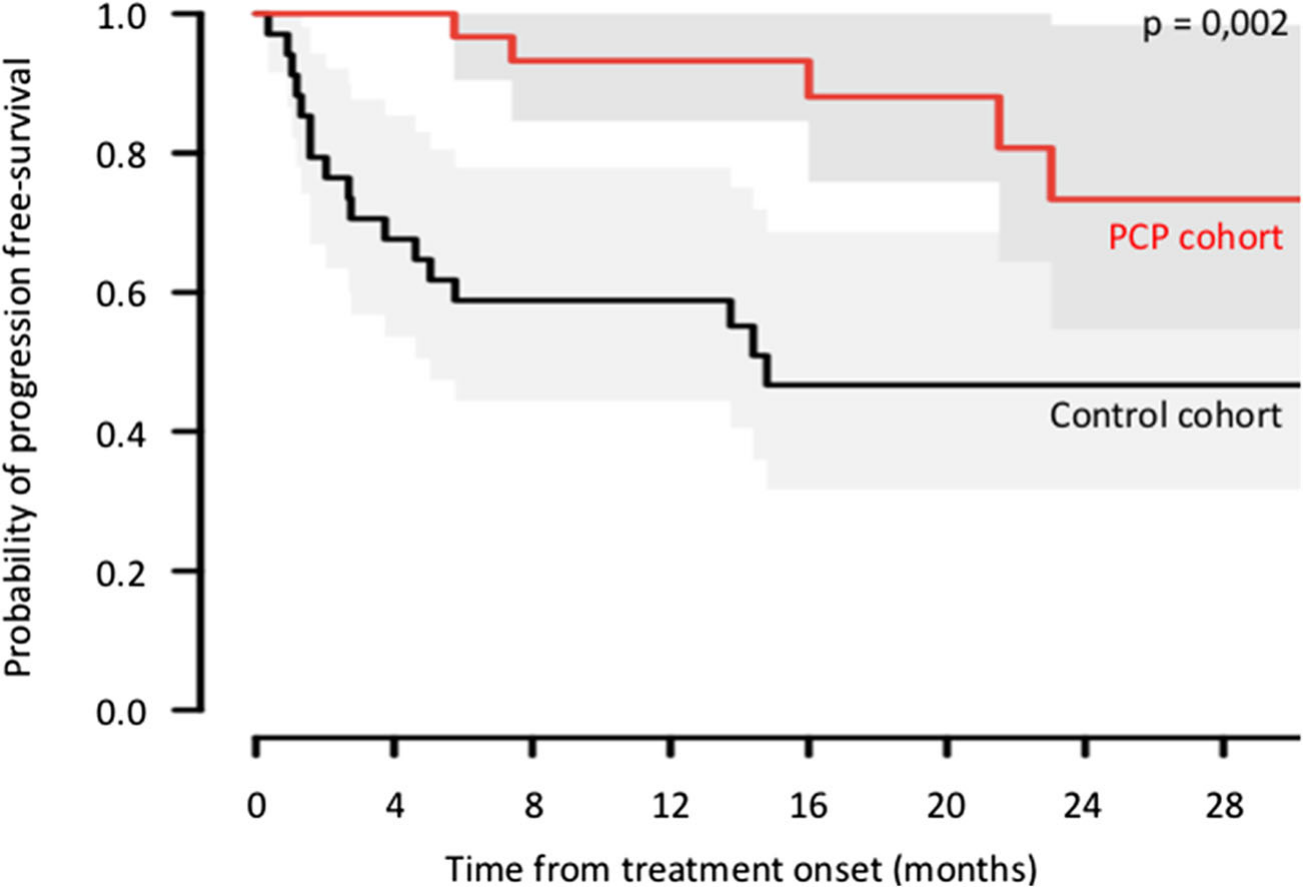
Comparison of progression-free survival outcomes on the matched basis. The Kaplan–Meier analysis is the probability of progression-free survival in the PCP group as compared with the control group. Median time for progression is not reached at 30 months in the PCP groups as compared with a median time for progression of 14 months in the usual group

Based on the matched sample, the effect of PCP on PFS was significant (HR = 0.26; 95% CI, 0.11–0.61; *p* = 0.002) (Table 2). Sensitivity analysis based on average scores from 30 imputed complete datasets of 155 patients did not markedly modify these results; 35 of the 42 treated patients could be matched on their score. Estimated treatment effect was slightly modified (Table 2). The median was not reached at 30 months as compared with a median duration of PFS of 14 months in the usual care group (Fig. 4).

**Table 2:** Estimates of the PCP effect on progression-free survival, overall survival, and time to treatment failure before and after matching on propensity scores

Outcome	Before matching		After matching	
	HR (95% CI)	*p* value	HR (95% CI)	*p* value
Primary outcome, progression free survival				
Complete cases	0.43 (0.22–0.83)	0.012	0.26 (0.11–0.61)	0.002
Multiple imputation	N/A	N/A	0.31 (0.13–0.75)	0.009
Secondary outcomes				
Overall survival	0.44 (0.20–1.00)	0.050	0.17 (0.05–0.58)	0.004
Time to treatment failure	0.41 (0.22–0.77)	0.005	0.26 (0.12–0.56)	0.0005

*HR* hazard ratio, *CI* confidence interval, *N/A* not applicable

**Fig. 5:**
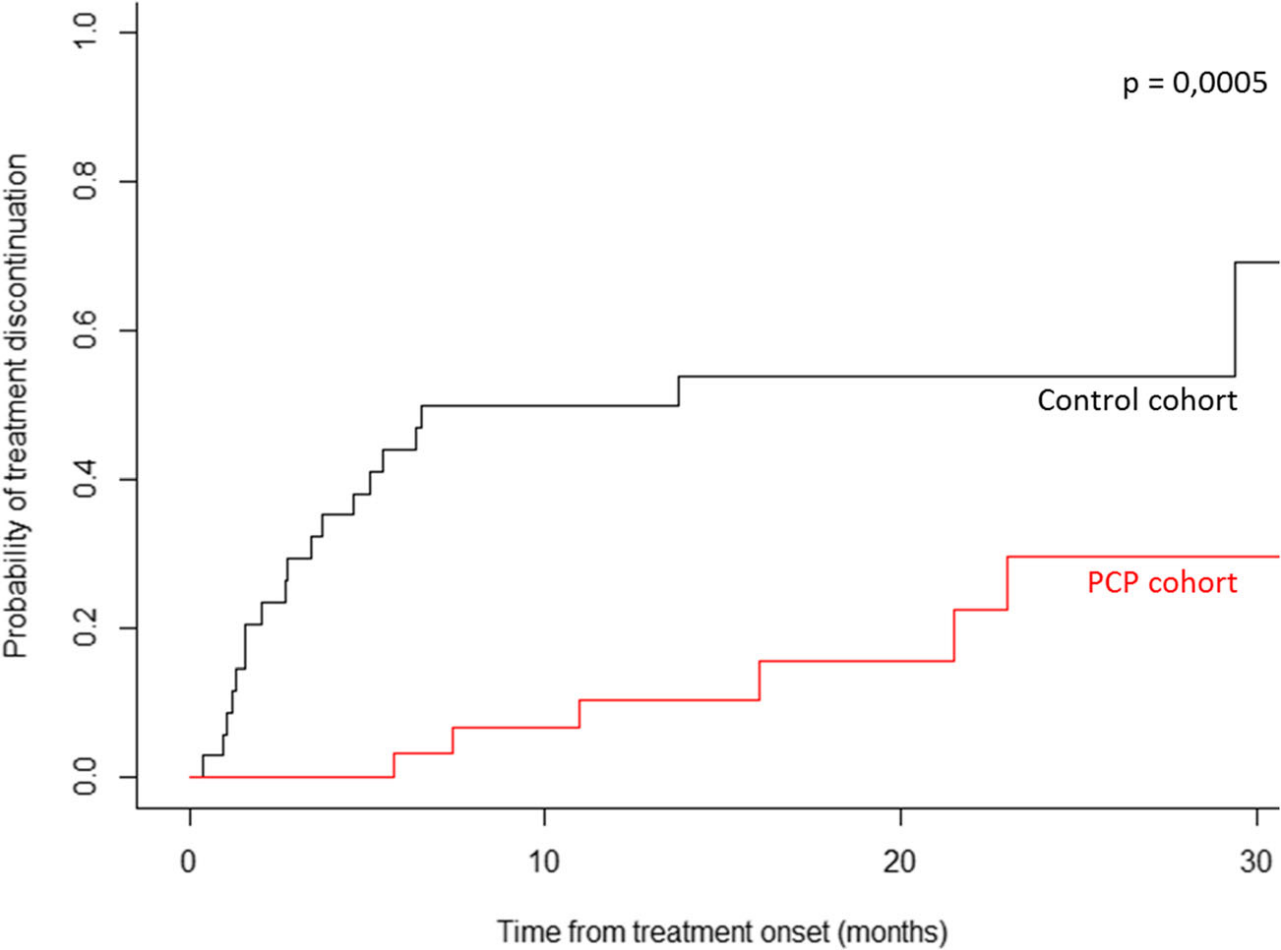
Comparison of time to treatment failure outcomes on the matched basis. The Kaplan–Meier analysis is the probability of time to treatment failure in the PCP group as compared with the control group. Median time for time to treatment failure is not reached at 30 months in the PCP groups as compared with a median time for progression of 27 months in the usual group. At 6 months, 90% of patients in the PCP group were still treated, as compared with 60% in the usual care group

**Fig. 6:**
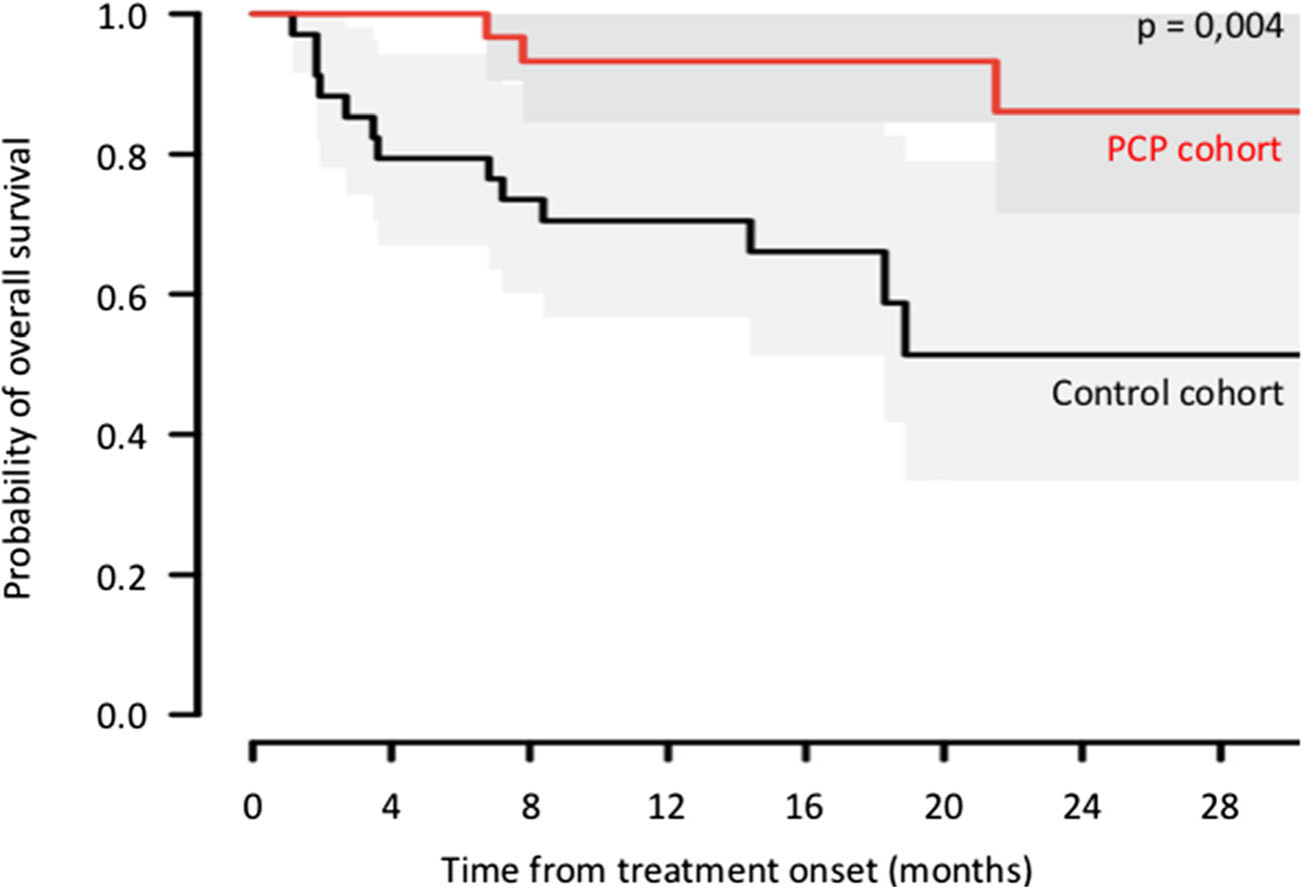
Comparison of overall survival outcomes on the matched basis. The Kaplan–Meier analysis is the probability of overall survival in the PCP group as compared with the control group. Median time for survival is not reached at 30 months in the PCP groups as compared with a median time for progression of 19 months in the usual group

Based on the matched sample, the effect of PCP was significant on time to treatment failure as well on overall survival (Table 2). The PCP significantly prolonged the duration of TTF, with a median not reached at 30 months as compared with a median duration of time to treatment failure of 27 months in the usual care group. At 6 months, 90% of patients in the PCP group were still treated (*n* = 38), as compared with 60% in the usual care group (*n* = 69) (Fig. 5). The PCP significantly prolonged the OS, with a median not reached at 30 months as compared with a median OS of 19 months in the usual care group (Fig. 6). Comparison of PFS, TTF, and OS outcomes on the original basis is presented on Supplementary materials.

Filling of the patient diary for self-evaluation of adherence was significantly more respected in PCP group than control group (Table 3). The adherence rate was higher for the PCP group, whether measured by self-evaluation, 99% vs. 92% (NS), or by calculation of the MPR, 99% vs. 90% (NS). Drug interactions were substantially similar between PCP and control groups at initiation, 1.0 and 0.83 DDI by patient respectively. After 1 month, patients in PCP had fewer DDIs (0.19, i.e., 81% reduction) while control patients had almost as many drug interactions as initiation (0.77, i.e., 7.2% reduction). The proportion of patients with at least one DDI decreased from 64 to 14% in the PCP group and from 56 to 49% in the control group.

**Table 3:** Effect of PCP on adherence and drug interactions

	PCP	Usual care	*p* value
Adherence			
Filling of the patient diary (self-evaluation) – no. (%)	34 (81)	51 (45)	0.004
Mean adherence (self-evaluation) – % (SD)	99 (0.7)	92 (23.1)	0.15
Mean adherence (medication possession ratio) – % (SD)	99 (2.3)	90 (25.8)	0.22
Drug interactions			
DDI by patient at initiation – mean (range)	1.0 (0–4)	0.83 (0–5)	0.38
DDI by patient at 1 month – mean (range)	0.19 (0–3)	0.77 (0–5)	< 0.0001

*PCP* pharmaceutical care program, *SD* standard deviation, *DDI* drug-drug interaction

### Safety outcomes

All 155 patients were evaluated for safety. The most common any grade AEs, defined as those observed in 10% or more in either group, were infections (15% control, 16% PCP), fatigue (10% control, 24% PCP), diarrheas (9% control, 20% PCP), hemorrhage events (9% control, 18% PCP), and muscle spasms (7% control, 13% PCP). Adverse events of any grade were less common in control than PCP patients (NS) (Table 4).

**Table 4:** Adverse events, serious adverse events, and ibrutinib dose adjustment

Adverse event of any grade	Event – no. (%)		Ibrutinib dose adjustment – no. ^a^	
	PCP	Usual care	PCP	Usual care
Fatigue	42 (24)	37 (10)	2	1
Diarrhea	34 (20)	36 (9)	1	0
Petechiae and bleeding	31 (18)	36 (9)	2	1
Infection and infestation	27 (16)	59 (15)	4	1
Muscle spasm	23 (13)	27 (7)	0	1
Cardiac disorder	7 (4)	21 (5)	1	0
Neutropenia	4 (2)	10 (3)	6	3
Thrombocytopenia	0	11 (3)	0	0
Adverse event of grade ≥ 3				
All	13 (8)	57 (15)	N/A	N/A
Neutropenia	4 (2)	8 (2)	N/A	N/A
Fatigue	3 (2)	8 (2)	N/A	N/A
Infection and infestation	2 (1)	20 (5)	N/A	N/A
Diarrhea	2 (1)	8 (2)	N/A	N/A
Cardiac disorder	1 (1)	6 (2)	N/A	N/A
Rash	1 (1)	0	N/A	N/A
Thrombocytopenia	0	3 (1)	N/A	N/A
Petechiae and bleeding	0	3 (1)	N/A	N/A
Serious adverse event				
Neutropenia	2 (1)	2 (1)	N/A	N/A
Cardiac disorder	0	2 (1)	N/A	N/A
Infection and infestation	0	1 (0)	N/A	N/A
Diarrhea	0	1 (0)	N/A	N/A

*PCP* pharmaceutical care program, *N/A* not applicable

^a^Dose reduction or suspension of ibrutinib treatment until event correction possibly followed by a dose reduction

Grade ≥ 3 AEs occurred in larger proportions for patients in control (15%) than PCP (8%) patients. The most common grade ≥ 3 AEs observed was infections (5% control, 1% PCP). Discontinuation of treatment owing to AEs did not occur in the PCP group and occurred for 25% patients in the control group (in orders of frequency, diarrhea, cardiac disorder, infections, fatigue, and arthralgia).

There have been more therapeutic adaptations of ibrutinib (treatment suspension and/or dose reduction) following side effects in the PCP group (2% control, 9% PCP).

## Discussion

This observational study aimed at evaluating the effects of a PCP in patients receiving ibrutinib, assuming that it could increase PFS by reducing dose reduction or treatment discontinuation. To our knowledge, results are the first to date showing a significant improvement of PFS and OS in patients treated by an oral chemotherapy because of a monitoring program, with a significantly delayed treatment failure and a reduction in severe toxicities.

Although the literature provides effective approaches to improving the management of OOA-treated patients, there is no single, formalized, and extrapolatable method yet. In 2013, the American Society of Clinical Oncology (ASCO) and the Oncology Nursing Society (ONS) added a specific component for monitoring of OOA to their safety recommendations [20]. It defined actions that the clinician must perform to educate the patient to adherence and AEs self-management: a treatment plan must be given to the patient, including information about the molecule as well as therapeutic goal. The clinician should provide additional written information regarding the dosing schedules, AEs key symptoms to look for and how to react to these symptoms, treatments to manage non-serious AEs, and possible DDIs. Furthermore, the clinician must assess the level of patient adherence at each consultation. The oncologist often has limited time during medical consultations. The use of other health professionals has proved the ability of multidisciplinary teams included pharmacists and nurses to achieve the expected results [13]. Some multidisciplinary programs involving pharmacists have shown ability to reduce the severity of adverse events and improve adherence [21].

Literature data associated non-adherence to ibrutinib with decreased rates of PFS [9]. Other OOAs in hematology have shown that adherence is the main factor of treatment efficacy [22]. We used the combination of two methods of measuring adherence. More expensive methods exist, such as electronic pill dispensers or blood tests, but they did not show superiority to detect poor adhesion [23]. Patients in our program had higher rates of adherence than the control cohort. This improvement is partly due to the educational component of the program. It depends on initial recommendations, ongoing evaluation and reminders from all stakeholders. Inclusion in the program allows the detection of poor compliance prospectively and its rapid correction. Indeed, patients in both cohorts were asked to self-assess their adherence. It was respected for less than half of the patients in the control group, limiting the ability to detect and correct poor adherence.

Our current study further demonstrates a significant improvement in tolerance of patients in PCP cohort. This is mainly the result of three components of the program: patient education to self-management of AEs, transition from hospital to community, and reduction of DDIs. There is evidence that patient education to self-management of AEs has a beneficial impact on tolerance [24]. In our study, instructions on ibrutinib toxicities were elaborated by a multidisciplinary team involving oncologists, pharmacists, infectiologists, biologists, and nutritionists. These recommendations were evidence-based medicine and concerned digestive, hematological, infectious, cardiovascular, and hemorrhagic events [25–27]. This led to institutional educational materials that were provided to patients in PCP at initiation of treatment. In our study, more low-grade adverse events have been reported in PCP group, which can be explained by improved detection thanks to the intervention. Indeed, the program provides telephone calls by clinical pharmacists and promotion of relations from hospital to community with specialist and general practitioners and community pharmacists. These measures showed that the detection of AEs was improved by 59% after 6 months in a pharmaceutical program of education and follow-up of onco-hematologic outpatients [28]. Improving the detectability of toxicities makes it possible to manage it before aggravation. Finally, the prophylaxis of toxicities also depends on the medication-related interventions, in particular when it corrects the DDIs responsible for an increase of ibrutinib in the plasma and prevents the consequent toxicities. At the first prescription of ibrutinib, proportion of patients in both PCP and cohort groups with DDIs were comparable to the literature [16]. The number of DDIs per patient in PCP group was divided by four at 1 month of treatment, lowering to less than 0.2 per patient. All DDIs could not be removed and this led in some cases to a decrease of ibrutinib dosage due to an inevitable association with a strong enzyme inducer.

Another outcome studied with a direct impact on the effectiveness of ibrutinib was the time to treatment failure. Our results have revealed a significantly increased TTF for patients included in the PCP. A real-world analysis has shown rate of ibrutinib discontinuations due to toxicities range from 12 to 32% [29]. In our control cohort, the rate of ibrutinib discontinuation attributable to side effects over the entire study period was 25% versus 0% in the PCP cohort. As reported in the literature, this can be explained by common dose reductions and temporary treatment interruptions to manage the toxicity early without stopping the treatment permanently [30]. Early detection of adverse effects that allow the multidisciplinary program for patients in PCP cohort lead to more suspensions of treatment, followed or not by a dose adjustment, as compared to the control cohort. Temporary cessations and dose decreases due to adverse events were commonly possible because it does not reduce the long-term efficacy of ibrutinib [10].

Our study has some limitations. First, it was a single-center study based on a sample size of 155. Second, the two groups were not constituted through randomization but depended on the oncologist decision, so that some selection bias may have occurred; it is likely that the program was mostly proposed to those patients with a past history of intolerance or of poor adherence, or at high risk of adverse events due to comorbidities. Therefore, we used propensity score matching to allow handling such a confounding-by-indication bias. Nevertheless, matching on propensity scores leads to unbiased estimation of treatment effects when certain assumptions hold, notably that all confounding covariates were observed and included in the propensity score; then, this implies the balance generated by propensity score matching leads to unbiased treatment effect estimates. However, we cannot exclude the possibility of unmeasured confounders. Thus, further multicenter randomized studies should confirm the benefits of such a PCP.

## Conclusion

Our program is consistent with the various recommendations concerning OOA. The individualization of the care pathway with adherence and tolerance monitoring, patient education for self-management, and coordination from hospital to community allow a better use of ibrutinib. A pharmaceutical care program provides a personalized environment for outpatient monitoring and control of the key risks associated with OOA. This study shows for the first time evidence that such a program improves adherence and tolerance with ibrutinib leading to longer treatment durations that are directly related to better PFS and OS. Our findings support the need of clinical pharmacists in the oral chemotherapy management.

## Electronic supplementary material

ESM 1 (TIFF 19.3 mb)

## Data Availability

The datasets generated during and/or analyzed during the current study are available from the corresponding author on reasonable request.
