# Supplementary figures and images for: Improved time to treatment failure and survival in ibrutinib-treated malignancies with a pharmaceutical care program: an observational cohort study
Source: Ann Hematol. 2020 Jun 1;99(7):1615–25. doi: 10.1007/s00277-020-04045-y (PMC7316844; doi:10.1007/s00277-020-04045-y)

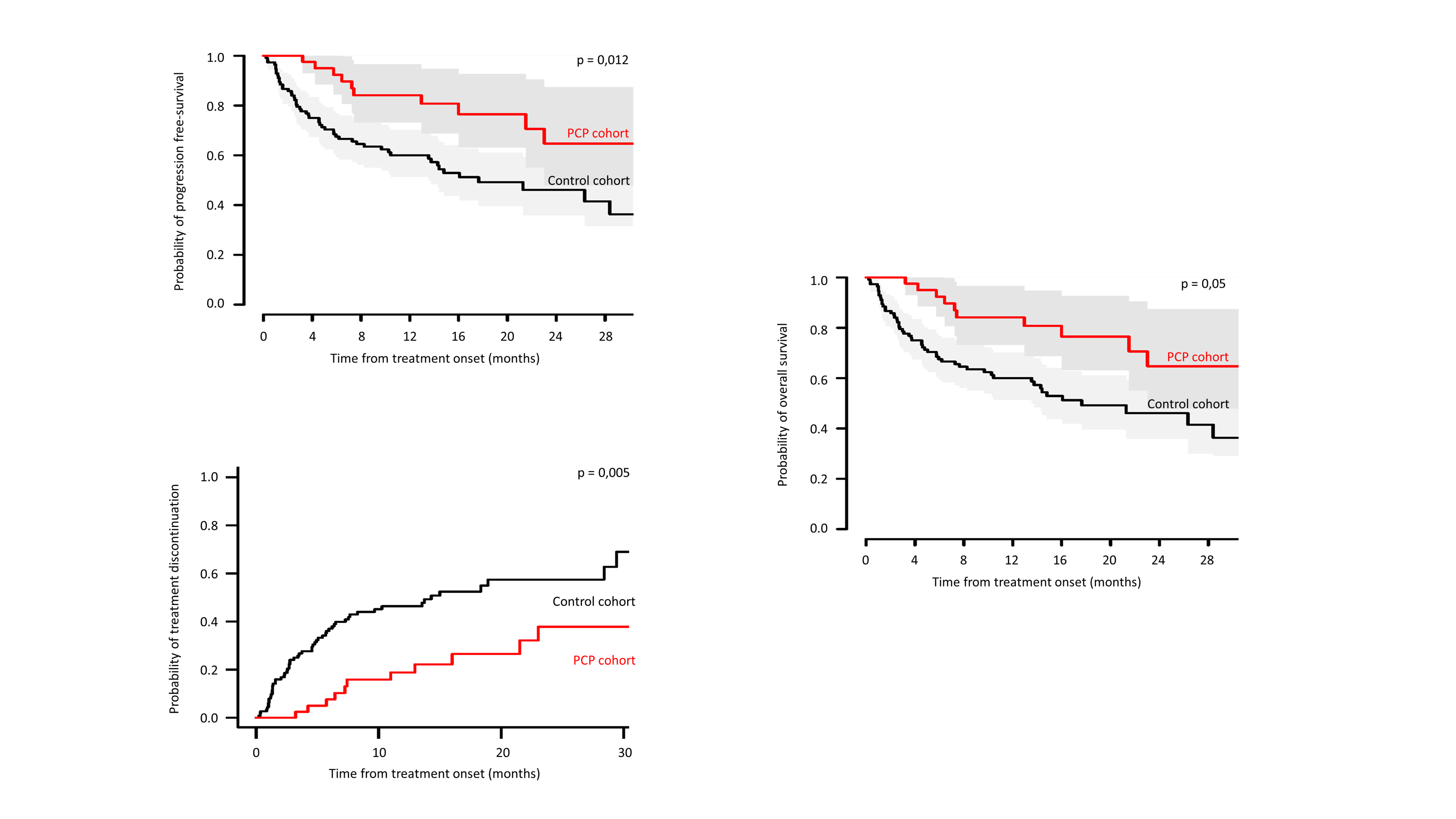

Supplement: Supplementary file 1 — (TIFF 19.3 mb) [file 277_2020_4045_MOESM1_ESM.tiff]
